# Reproductive studies on the carpet clam *Paphia textile* (*Paratapes textilis*) (Gmelin 1791) (Family: Veneridae): a guide of aquaculture management along the Egyptian coasts of the Red Sea and Suez Canal

**DOI:** 10.1186/s40850-023-00179-4

**Published:** 2023-09-07

**Authors:** Marwa I. Farghaly, Tamer El-Sayed Ali, Hanan M. Mitwally, Fatma A. Abdel Razek

**Affiliations:** 1 Oceanography Department, Faculty of Science, Alexandria University, Alexandria, Egypt; 2 National Institute of Oceanography and Fisheries (NIOF), Alexandria, Egypt

**Keywords:** Reproductive cycle, Sex ratio, Spatial distribution, Temporal distribution, Environmental parameters, Condition index, Gonad index, Maturity size

## Abstract

**Background:**

Most aquatic biota's reproductive biology and life cycle are essential to the sustainable management and development of coastal ecosystems and aquaculture. The bivalve *Paphia textile* (Gmelin 1791), also known as *Paratapes textilis*, has an economic value in Indo-Pacific waters, including the Red Sea and the Suez Canal lakes, the Egyptian coasts. However, *P. textile* suffers from extensive fishing and exploitation.

**Aim:**

The present work aims to study the *Paphia textile's* reproductive cycle on the Egyptian coasts of the Red Sea for the first time. It helps to manage and develop the coastal ecosystems and aquaculture.

**Methodology:**

Samples were collected monthly from two saline lakes in the Suez Gulf from December 2019 to November 2020. As part of the comprehensive research study, sex ratio, condition index, sexuality, histological analysis of gonads, shell size, and gonad index were used to investigate the reproductive cycle.

**Results:**

The results reveal a male-biased sex ratio, possibly due to anthropogenic stressors. The *Paphia textile* is dioecious. No hermaphrodite cases were observed in the studied specimens. The condition index in winter and spring indicates periods dominated by mature individuals. Five reproductive maturity stages were assigned for both *P. textile* males and females. Due to the simultaneous development of several developmental stages monthly throughout the sampling year, warm water may be responsible for non-sequential gametogenic cycles. As measured environmental parameters correlate with maturity stages, temperature, salinity, and chlorophyll *a* play important role in gonad growth. The size at first sexual maturity at which 50% of the *Paphia textile* population reached maturity ranged from 28.60 to 31.50 mm for females, and between 31.70 and 34.10 mm for males. As the gonad index increases during the ripe stages, this index decreases during the resting, spawning, and spent phases.

**Conclusions:**

The findings suggest the most suitable temperature for aquaculture spawning is between 20 °C and 30 °C in subtropical waters. Fishing should generally be prohibited at sizes less than 28.60 mm for better management and sustainability of this valuable aquatic resource on the Egyptian coasts of the Red Sea.

**Supplementary Information:**

The online version contains supplementary material available at 10.1186/s40850-023-00179-4.

## Background

*Paphia textile* (Gmelin, 1791), or its relevant synonyms *Paratapes textilis* and *Venus textile* (Gmelin 1791), which refers to the family name Veneridae or venus clam (https://www.ciesm.org/atlas/Paphiatextile.html, 2005), and the recent update of marine worm species, WORMS, 2023 (https://www.marinespecies.org/aphia.php?p=taxdetails&id=214508) is a filter feeder mollusk, widely distributed in warm water and inhabited a wide range of salinities at the continental shelf of the Indo-pacific waters (including the Red Sea and the Gulf of Suez, CIESM, 2005). Most molluscan fauna dominates sandy substrates, and its typical size ranges between 45–55 mm, while its shell lengths are vary between 18.20 and 63.40 mm [1]. It is characterized by a pale yellowish-white shell, ornamented with attractive patterns with a smooth glossy surface [2]. *Paphia textile* has great economic value as an affordable food with a high nutritional content as most seafoods [3–5]. In Egypt, the annual production of *P. textile* varies from 854 to 8910 tons per year, which accounted for more than 70% of the total production of Bivalvia in Ismailia city (5). Bivalvia fishing gear consists of boat rakes, hand rakes, and diving for bivalves in Ismalia.

Many studies documented the occurrence of *P. textiles in* Egyptian waters of the Red Sea and Mediterranean [6, 7] or used it in risk assessment procedures [8]. However, the first intensive biological studies of this carpet clam were done in 2019–2020 [1], which found that the species undergoes high mortality and exploitation along the Suez Canal, Egypt, and recommended better management of this valuable marine resource.

The study of the reproductive cycle of the present clam species is necessary to sustain their health, harvesting seasons, and future farming and management [9]. The gametogenesis and spawning are genetically controlled based on surrounding environmental factors and food availability [10, 11]. The histological analysis of each sex’s gonadal maturity is a well-documented assessment of the reproductive cycle [12, 13]. The development of gonad maturity was classified into five or six stages according to the observed components in most follicles [14–17].

For the successful rear of the carpet shell clam under controlled conditions, it is essential to study reproductive biological characteristics, such as the sex ratio, condition index, gonad index, and stages of gonadal maturity. The sex ratio or sex determination is used as a bioindicator for environmental health, economic and nutritional values, conservation, maintenance, and species restocking [18]. The sex ratio provides valuable information on the proportion of males to females and their ability to undergo sex reversal in the population for a future artificial breeding program [19]. The condition index is essential in defining the Bivalvia health status through the meat quality and accordingly predicting the harvest timing of the farmed species [19]. The gonad index is a numerical ascending score ranging in molluscan studies from 0 to 4, 5, or higher [20]. In some studies, the highest score is three, which indicates the most developed gonads (the ripe stage), and the zero or one score indicates the gonads in the resting and spent stages, respectively [21–23]. It is used to estimate the relative proportions of male and female individuals in different reproductive stages [24]. Size at first sexual maturity is a reproductive metric indicator in which 50% of the population reached maturity at a given shell length [25]. Moreover, fully mature individuals at a given size can be used in brood stock for future hatchery programs [19].

Our knowledge regarding the *P. textile* reproductive cycle and biology is scarce, despite this clam’s economic value. Several well-known studies were conducted in the Philippines [26, 27]. Thus, the present work aims to provide an intensive investigation of *P. textile* reproductive biology on the Egyptian coasts of the Suez Canal. Two essential commercial fisheries, Timsah Lake and Great Bitter Lake, were chosen for the present clam sampling. Then, to identify significant spatial distribution alongside the temporal variations between the studied areas and throughout the sampling year. The information gained from this study is recommended knowledge for better planning and management of Bivalvia resources and enhances the future of the bivalves’ aquaculture industry.

## Methodology

### Study areas and sampling locations

#### Timsah lake (TL)

**Fig. 1:**
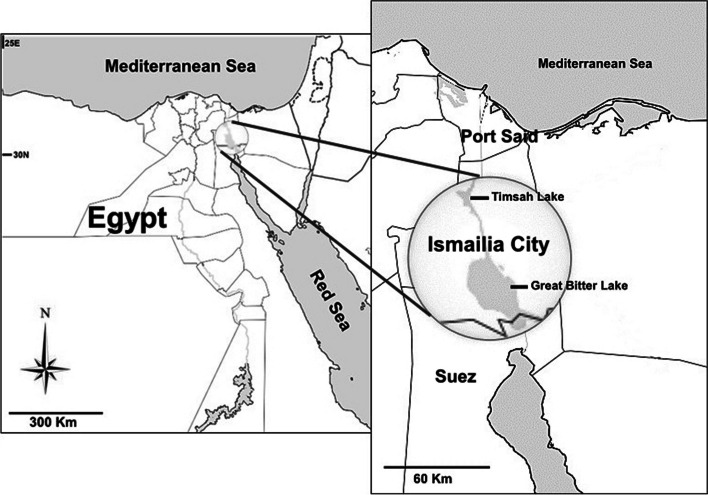
Study sampling sites along the Suez Canal, Egypt. Site I is in Timsah Lake, and Site II (Fayed City) is in Great Bitter Lake. This map was originally implemented using the interactive map maker website (https://mapmaker.nationalgeographic.org/map)

Timsah Lake is shallow large water body located at Ismalia city between the south and north boundaries of Suez and the Port Said (Suez Canal cities), respectively, at 30° 32′ 58′′—30° 35′ 29′′ N and longitude 32° 15′ 36′′—32° 18′ 23′′ E. It undergoes a wide range of saline water (14–40‰) [28], due to receiving different sources of waters. The maximum length, width, surface area, and depth of the lake are 4.55 km, 4.22 km, 9.1 km^2^ and 13 m, respectively. The present clam sampling site is on the south lake’ border where the commercial fishing occurs (Map, Fig. 1). The map of the current study is original, implemented using the interactive map maker free website https://mapmaker.nationalgeographic.org/map.

#### Great bitter lake (GBL)

Great Bitter Lake is located between 30° 13′ 15′′ and 30° 24′ 55′′ N and 32° 18′ 15′′ and 32° 28′ 31′′ E. It is hypersaline, as its salinity exceeds 41‰, but is less than 44.6 ‰ [29]. It covers 85% of the Suez Canal area. The lake’s maximum length, width, surface area, and depth are 63 km, 13 km, 194 km^2^, and 23 m. Fayed City is the site of the present study of clam species (Map, Fig. 1).

#### Specimen collection and metrics

The studied *P. textile* clam specimens were collected monthly from TL and GBL from December 2019 to November 2020. A total of 15.000 clam specimens were collected at 5 m depth from the well-known commercial catches in Ismailia city, Egypt, 8465 (TL) and 6467 (GBL). The samples were taken randomly from the most popular commercial sites at the south border of TL and Fayed site (GBL), as the scuba diving covered an area of relatively 50 m^2^. After sampling, the specimens were kept in pre-labeled net bags and transferred to the invertebrate laboratory. The specimens were sorted into 2-mm class intervals. Representative subsamples of each sorted class interval were selected for further analysis. A morphometric study was carried out on the selected specimens. Shell length (SL), shell width (SW), and shell inflation (SI) was measured using Vernier calipers to the nearest 0.05 mm. The SL and SW measurements were taken across the specimen’s longest and widest parts. The clam’s body weight was measured using a digital balance to the nearest 0.01 g. The data from the morphometric measurements were analyzed using linear regression analysis and published in our previous paper [1]. The morphological diagram was added herein as Appendix 2.

#### Environmental parameters

Environmental parameters were measured seasonally during 2020, the pandemic year. Water temperature, pH, salinity, and dissolved oxygen (DO) were determined using the portable Hydrolab (YSI 556). Chlorophyll *a* concentration was assessed according to the standard methods APHA [30].

#### Sex ratio

To estimate the sex ratio, a subsample of relatively 100–130 individuals was taken monthly from TL and GBL population. In total, more than 1200 specimens were investigated, but less than 1300 at each location. The sex ratio was determined by identifying each clam’s sex via gonad smear preparation under a light compound microscope (Olympus CX31).

#### Condition index

The condition index (CI) was estimated based on the Davenport and Chen following equation [31]. A total of 600–720 clam individuals were morphometrically examined throughout the whole study period at both sampling sites. Both shell and flesh were dried separately at 60 °C (OF-01E/ JEIO TECH) for 48 h after removing the soft tissues from their shells.

$$\mathrm{CI}= \left(\mathrm{dry\,flesh\,weight}\div \mathrm{dry\,shell\,weight}\right) \times 100$$

### Histological analysis

#### Tissue preparation

The standard bivalve mollusk histological tissue preparation was used [32]. Clam soft tissues were cut longitudinally into two halves after removing their shells and samples of gonads were separated for transverse section preparation. The samples were fixed in Davidson’s fixative solution [33] for 24 h and then immersed in a 70% alcohol solution. Next, tissues were dehydrated in ascending ethanol concentrations from 70–100%, cleared with xylene, and embedded in paraffin wax. Finally, all specimens were cut at 5 µm transverse sections with a rotary microtome (Leica RM2235), mounted on clean glass slides, and stained with hematoxylin–eosin dye for further microscope examination. The gonadal developmental stages of each specimen were examined using objective lenses, 4x, 10x, and 20 x, under a light compound microscope (Olympus CX31). Photographs were taken from each stage using the TOUPCAM TM UCMOS 03100KPA camera. Through the study, 600 female and 360 male specimens were used for histological analysis from TL and GBL.

#### Determination of the gonadal maturity stages

A modified scale was applied to assign a clam’s sex into one of the observed reproductive stages [14]. The clam reproductive stage, for both sexes, is assigned based on follicle appearance. Many developmental components can be observed in each specimen. The assignment of the reproductive stage was based on the ratio of the dominant viewed components. For each stage of development, a table was created comparing the histological differentiation of males and females.

#### Clam size at first sexual maturity

The percentage (%) of mature clams was plotted against shell length (SL). In the aquaculture industry, mature clams are carefully selected at stage III (ripe) or above IV (spawning), and V (spent) stages are retained [34]. The length at which 50% of the clam population is sexually mature (SM_50_) was calculated by fitting a logistic curve to the proportion of mature individuals vs. SL following the size at maturity (SAM) model illustrated according to the following equation [25]:

$$\mathrm{PL}= \left(1 + {\mathrm{e}}^{-\mathrm{ln }\left(19\right) \left(\mathrm{L}- {\mathrm{L}}_{50}\right)/\left({\mathrm{L}}_{95}- {\mathrm{L}}_{50}\right)}\right)$$

where P_L_ is the percentage of mature clams in length class L; L_50_ and L_95_ are the lengths at which 50% and 95% of clams in that length class are mature.

#### Gonad index scores

The gonad index is calculated based on the number of individuals that have simultaneously the same developmental stage multiplied by the proposed rank (score) of this stage according to the [35] schemes cited in [21] and divided by the total number of investigated individuals per month.

$$\mathrm{GI}=\frac{\sum\mathrm n\mathrm o.\,\mathrm o\mathrm f\,\mathrm i\mathrm n\mathrm d\mathrm i\mathrm v\mathrm i\mathrm d\mathrm u\mathrm a\mathrm l\mathrm s\,\mathrm f\mathrm r\mathrm o\mathrm m\,\mathrm e\mathrm a\mathrm c\mathrm h\,\mathrm d\mathrm e\mathrm v\mathrm e\mathrm l\mathrm o\mathrm p\mathrm m\mathrm e\mathrm n\mathrm t\mathrm a\mathrm l\,\mathrm s\mathrm t\mathrm a\mathrm g\mathrm e\,\ast\,\mathrm s\mathrm t\mathrm a\mathrm g\mathrm e\,\mathrm n\mathrm u\mathrm m\mathrm e\mathrm r\mathrm i\mathrm c\mathrm a\mathrm l\,\mathrm r\mathrm a\mathrm n\mathrm k\mathrm i\mathrm n\mathrm g}{\mathrm T\mathrm o\mathrm t\mathrm a\mathrm l\,\mathrm n\mathrm o.\,\mathrm o\mathrm f\,\mathrm i\mathrm n\mathrm d\mathrm i\mathrm v\mathrm i\mathrm d\mathrm u\mathrm a\mathrm l\mathrm s\,\mathrm i\mathrm n\mathrm v\mathrm e\mathrm s\mathrm t\mathrm i\mathrm g\mathrm a\mathrm t\mathrm e\mathrm d\,\mathrm i\mathrm n\,\mathrm e\mathrm a\mathrm c\mathrm h\,\mathrm s\mathrm a\mathrm m\mathrm p\mathrm l\mathrm i\mathrm n\mathrm g\,\mathrm m\mathrm o\mathrm n\mathrm t\mathrm h}$$

There were six stages in Gosling's concept, ranging from 0 at the resting stage to 5 at the ripe stage. The scores 1, 2, 3, and 4 were assigned to spent, partially spawned, early active, and late active, respectively. Based on the histological studies of the gonad tissues in the current study, the gonad scores were slightly modified to five stages instead of six. The early and late stages were merged and assigned as the developing stage with a score of 3. Accordingly, the resting, developing, and ripe stages have numerical scores of 0, 3, and 4. The spawning and spent stages were ranked 2 and 1, respectively.

$$\mathrm{The}\;\mathrm{applied}\;\mathrm{GI}\;\mathrm{equation}\;\mathrm{is}\;\mathrm{GI}\;=\;\frac{\mathbf\Sigma\boldsymbol\;\mathbf{no}\boldsymbol.\boldsymbol\;\mathbf{of}\boldsymbol\;\mathbf{individuals}\boldsymbol\;\mathbf{from}\boldsymbol\;\mathbf{each}\boldsymbol\;\mathbf{developmental}\boldsymbol\;\mathbf{stage}\boldsymbol\;\boldsymbol\ast\boldsymbol\;\mathbf{stage}\boldsymbol\;\mathbf{numerical}\boldsymbol\;\mathbf{ranking}}{\mathbf{Total}\boldsymbol\;\mathbf{no}\boldsymbol.\boldsymbol\;\mathbf{of}\boldsymbol\;\mathbf{individuals}\boldsymbol\;\mathbf{investigated}\boldsymbol\;\mathbf{in}\boldsymbol\;\mathbf{each}\boldsymbol\;\mathbf{sampling}\boldsymbol\;\mathbf{month}}$$

### Statistical analysis

A classical Two-way ANOVA was used to test for significant differences in the mean raw data of different environmental parameters (temperature, pH, salinity, DO, and the log data of chlorophyll *a* concentration) between two categorical factors (locations and seasons), as well as their interactive effect, at α ≤ 0.05. The chi-square test for association (χ2) was applied to test if sex ratios would deviate from the standard 1:1 ratio. The analysis was applied monthly at each study location. The null hypothesis is that there are no significant differences between the sex ratio and the standard value of 1:1. The analysis was done according to Corder and Foreman [36] based on the chi-square equation:

$$\upchi 2=\sum \left(\frac{\left({\mathrm{O}}_{\mathrm{i}}-{\mathrm{E}}_{\mathrm{i}}\right)}{{\mathrm{E}}_{\mathrm{i}}}\right)$$

where O_i_ and Ei are the observed and expected numbers of each sex. Pearson chi-square values were considered significant at α ≤ 0.05.

The normality and homogeneity of variances in the data of chlorophyll *a*, condition index, maturity stages, and gonad indices were tested using histogram figures. Data of chlorophyll *a*, maturity stages I, II, IV, and V, deviated from the normal distribution and were square root transformed. The transformation step leads the data to be more normally distributed. A two-way ANOVA was performed to test for significant differences in the mean raw data of the CI between the sampling locations and months. The mean squared root transformed maturity stage data were tested for significant differences between sampling locations, sex, throughout the sampling period, and their interactions using a three-way ANOVA. A Tukey test was performed to test for significant differences between pairs of categorical factors (locations, sex, and months). The Pearson correlation coefficient was applied to test for significant correlations between the square root transformed maturity stage data and the raw data of the environmental parameters. The three-way ANOVA and the Tukey test were used to test for significant differences in the mean raw data of the GI between locations, sex, within months, and their interactions at the α ≤ 0.05. Statistical analyses were performed using the general linear model (GLM) procedure in SYSTA (8.0) software [37]. Using GLM on transformed data weakened the orthogonality of the survey studies [38].

## Results

### Environmental parameters

**Fig. 2:**
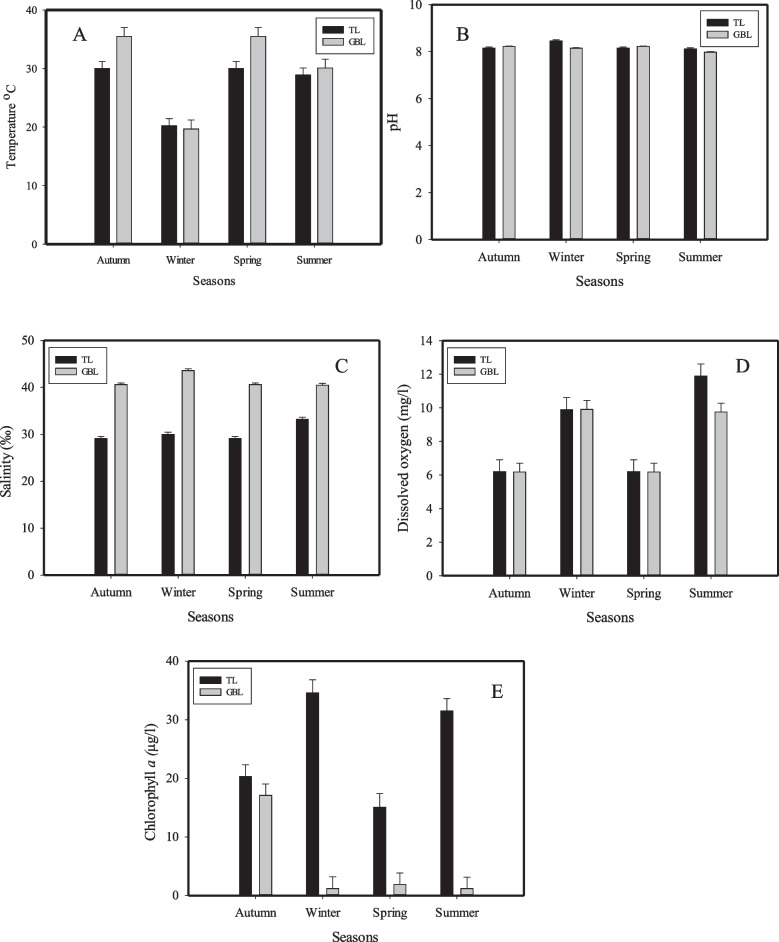
Seasonal averages of environmental parameter distribution in TL and GBL from December 2019 to November 2020. Abbreviations: TL = Timsah Lake, GBL = Great Bitter Lake

Water temperature (^◦^C) ranged from 19.7 ^◦^C (winter) to 35.5 ^◦^C (spring and autumn) and had higher records at GBL than that at TL during all seasons, except for winter (Fig. 2A). Mean water temperature significantly varied within seasons (*P* = 0.025, Table 1). Neither the locations nor the interaction effects between locations and seasons showed significant differences in water temperature. Temperature data were significantly different in winter compared to autumn and spring (Tukey test, Table 2). The highest and lowest values of the pH data were detected in winter (~ 8.5) in TL, and summer (~ 8.0) in GBL, respectively (Fig. 2B). Data analysis revealed no significant variations among locations, seasons, and their interactions. Salinity distribution fluctuated seasonally between 29.1‰ to 33.2‰ and from 40.5‰ to 43.6‰ at TL and GBL, respectively. Results of salinity distribution indicated normal and hypersaline patterns in TL and GBL, respectively (Fig. 2C). The mean salinity data revealed significant differences (*P* = 0.004) between the study locations. However, it showed no significant seasonal differences between seasons (Table 1). The range of dissolved oxygen data (DO mg/L) was between 6.2–11.9 mg/L and 6.18–9.91 mg/L, respectively, at TL and GBL (Fig. 2D). Mean DO data showed significant seasonal variations (*P* = 0.016) and data between the study locations were not meaningful (Table 1). A Tukey test indicated significant differences in DO data in autumn and spring vs. summer and winter (Table 2). The highest chlorophyll *a* concentrations were 34.6 µg/l and 17.1 µg/l, respectively, at TL and GBL, whereas the lowest concentrations were 15.1 µg/l and 1.2 µg/l, respectively, at TL and GBL (Fig. 2E). The chlorophyll *a* distribution pattern between TL and GBL was significantly different (*P* = 0.02, Table 1). The visual investigation of chlorophyll *a* data showed variations among seasons (Fig. 2E), but they were not significantly different (F = 6.12 and *P* = 0.144, Table 1).

**Table 1:** Results of two-way ANOVA based on mean raw data of temperature, pH, salinity, DO and log transformed data of Chlorophyll *a* concentration between locations, within seasons, and their interaction at α ≤ 0.05

Parameters	Source	SS	df	MS	F-ratio	*P*
**Temperature**	Location	16.965	1	16.965	3.595	0.154
	Season	219.126	3	73.042	15.479	**0.025**
	Location*Season	14.156	3	4.719	0.080	0.968
**pH**	Location	0.011	1	0.011	0.659	0.476
	Season	0.065	3	0.022	1.366	0.402
	Location*Season	0.048	3	0.016	0.841	0.538
**Salinity** **(‰)**	Location	240.901	1	240.901	69.001	**0.004**
	Season	7.804	3	2.601	0.745	0.593
	Location*Season	10.474	3	3.491	0.056	0.980
**DO** **(mg/l)**	Location	0.594	1	0.594	1.038	0.383
	Season	35.708	3	11.903	20.789	**0.016**
	Location*Season	1.718	3	0.573	0.063	0.977
**Chlorophyll *a*** **(µg/l)**	Location	12.62	1	12.62	48.90	**0.020**
	Season	4.74	3	1.58	6.12	0.144
	Location*Season	10.01	3	3.34	2.43	0.242

*Abbreviations*: *DO* Dissolved oxygen, *SS* Sum squares, *df* Degree of freedom, *MS* Mean square, *F-ratio* F statistic, *P* Probability at α ≤ 0.05 (in bold values)

**Table 2:** Results of the posteriori hoc comparison analysis Tukey test based on the mean raw data of temperature and DO among seasons at α ≤ 0.05

**Seasons**	Temperature		DO	
	**T**	***P***	**T**	***P***
**Autumn-Spring**	< 0.001	1.000	< 0.001	1.000
**Autumn-Summer**	-3.250	0.533	4.635	**0.026**
**Autumn–Winter**	-12.775	**0.029**	3.715	**0.048**
**Spring–Summer**	-3.250	0.533	4.635	**0.026**
**Spring-Winter**	-12.775	**0.029**	3.715	**0.048**
**Summer–Winter**	-9.525	0.064	-0.920	0.659

*Abbreviations*: *DO* Dissolved oxygen, *P* Probability at α ≤ 0.05 (the bold values), *T* The least mean square difference

### Sex ratio

The total number of individuals whose sex was assigned was 1242 and 1228 at TL and GBL, respectively. The male contribution was 55.64% and 56.03%, whereas the percentage of females was 44.36% and 43.97% at TL and GBL, respectively (Table 3). The overall sex ratios (M: F) were 1: 0.8 in TL and 1: 0.78 in GBL. Results of Chi-square (χ2) rejected the null hypothesis and revealed deviations in the ratios from the standard value (1:1), as the χ2 values were 15.78 and 17.84 at *P* < 0.001 at TL and GBL, respectively. A significant monthly deviation in the sex ratio was recorded at both TL and GBL (Table 3). Excepting July at GBL, the data showed a male-skewed distribution. A graph of Chi-square monthly distribution results is shown in Appendix 1.

**Table 3:** Results of the sex ratios and Chi-square (χ2) values of the collected samples of *Paphia textile* from TL and GBL from December 2019 to November 2020

Month	Male		Female		Total		Sex ratio (M: F)		χ2		*P*	
	**TL**	**GBL**	**TL**	**GBL**	**TL**	**GBL**	**TL**	**GBL**	**TL**	**GBL**	**TL**	**GBL**
**Dec**	82	77	67	53	149	130	1: 0.82	1: 0.69	1.510	**4.431**	0.219	**0.0353**
**Jan**	68	69	32	31	100	100	1: 0.47	1: 0.45	**12.960**	**14.440**	** < 0.001**	** < 0.001**
**Feb**	63	72	37	27	100	99	1: 0.59	1: 0.38	**6.760**	**20.454**	**0.009**	** < 0.0001**
**Mar**	58	45	42	55	100	100	1: 0.72	1: 1.22	2.560	1.000	0.111	0.317
**Apr**	49	65	51	35	100	100	1: 1.04	1: 0.54	0.040	**9.000**	0.841	**0.003**
**May**	65	66	30	34	95	100	1: 0.46	1: 0.52	**12.894**	**10.240**	** < 0.001**	** < 0.01**
**Jun**	51	47	48	53	99	100	1: 0.94	1: 1.13	0.091	0.360	0.763	0.549
**Jul**	42	38	59	61	101	99	1: 1.4	1: 1.61	2.861	**5.343**	0.091	**0.021**
**Aug**	53	51	45	49	98	100	1: 0.85	1: 0.96	0.653	0.040	0.419	0.841
**Sep**	49	49	51	51	100	100	1: 1.04	1: 1.04	0.040	0.040	0.841	0.841
**Oct**	54	58	46	42	100	100	1: 0.85	1: 0.72	0.640	2.560	0.423	0.111
**Nov**	57	51	43	49	100	100	1: 0.75	1: 0.96	1.960	0.040	0.161	0.841
**Total**	691	688	551	540	1242	1228	1: 0.8	1: 0.78	**15.781**	**17.837**	** < 0.0001**	** < 0.0001**

*Abbreviations*: *TL* Timsah lake, *GBL* Great Bitter Lake

*P* Probability at α ≤ 0.05

### Condition index

**Fig. 3:**
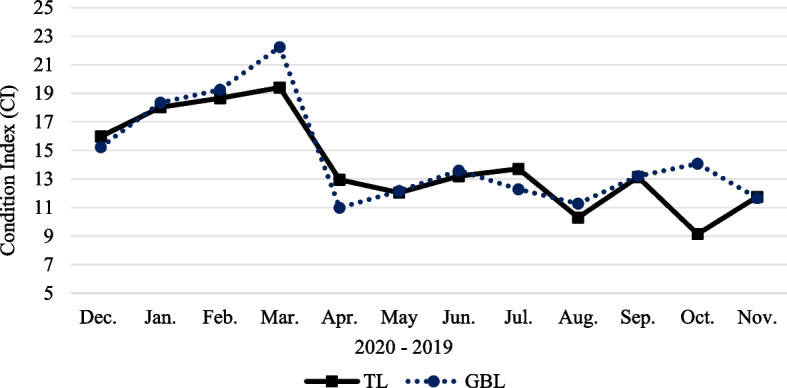
Condition index (CI) of *Paphia textile* results at TL and GBL from December 2019 to November 2020. Abbreviations: TL = Timsah Lake; GBL = Great Bitter Lake

The CI values of *P. textile* at TL and GBL tracked each other during the sampling period, except for July and October (Fig. 3). The range of *P. textile* CI at TL was between 9.15 ± 1.23 (October) and 19.41 ± 3.22 (March). While in GBL, the highest and lowest values were recorded in March (22.24 ± 10.99) and April (10.99 ± 1.61), respectively. There were no significant variations in the mean CI values between TL and GBL. However, the mean data for CI differed significantly among months (Table 4). The statistically significant varied data were scattered and detected during January and February (winter) vs. April and May (spring), August (summer), and October and November (autumn), as was shown by the least mean difference (T) in Table 5. The* P* values for January's mean differences were 0.017, 0.019, 0.005, 0.011, and 0.012, respectively, and February's least different means were at *P* = 0.007, 0.008, 0.036, 0.023, 0.002, 0.027, 0.005, and 0.005, respectively, vs. April, May, June, July, August, September, October, and November (Table 5). Similarly, March (spring) had significantly different mean data for CI from April, May, June, July, August, September, October, and November (*P* = 0.001, 0.001, 0.004, 0.003, 0.0001, 0.003, 0.001, 0.001, respectively, Table 5).

**Table 4:** Results of a two-way ANOVA of mean condition index of *Paphia textile* between the locations and throughout the study months from December 2019 to November 2020

Source	SS	df	MS	F-ratio	*P*
Location	1.512	1	1.512	0.891	0.366
Months	242.814	11	22.074	13.004	** < 0.0001**

*Abbreviations*: *SS* Sum squares, *df* Degree of freedom, *MS* Mean square, *F-ratio* F statistic

* P* Probability at α ≤ 0.05 where the bold values indicate substantial differences

**Table 5:** Results of Tukey test of the mean condition index of *Paphia textile* throughout the study months from December 2019 to November 2020

Months	CI		Months	CI		Months	CI	
	**T**	***P***		**T**	***P***		**T**	***P***
Dec.-Jan	2.581	0.698	Feb.-Apr	6.982	**0.007**	Apr.-Nov	-0.266	1.000
Dec.-Feb	3.347	0.390	Feb.-May	-6.852	**0.008**	May-Jun	-1.304	0.994
Dec.-Mar	5.226	0.053	Feb.-Jun	-5.548	**0.036**	May-Jul	-0.895	1.000
Dec.-Apr	3.636	0.297	Feb.-Jul	-5.958	**0.023**	May-Aug	1.310	0.994
Dec.-May	-3.506	0.336	Feb.-Aug	8.162	**0.002**	May-Sep	1.064	0.999
Dec.-Jun	-2.202	0.842	Feb.-Sep	-5.788	**0.027**	May-Oct	-0.492	1.000
Dec.-Jul	-2.611	0.685	Feb.-Oct	-7.345	**0.005**	May-Nov	-0.396	1.000
Dec.-Aug	4.816	0.083	Feb.-Nov	-7.248	**0.005**	Jun.-Jul	0.409	1.000
Dec.-Sep	-2.442	0.754	Mar.-Apr	8.856	**0.001**	Jun.-Aug	2.614	0.684
Dec.-Oct	-3.998	0.205	Mar.-May	-8.726	**0.001**	Jun.-Sep	-0.240	1.000
Dec.-Nov	-3.902	0.227	Mar.-Jun	7.421	**0.004**	Jun.-Oct	-1.796	0.946
Jan.-Feb	-0.765	1.000	Mar.-Jul	7.831	**0.003**	Jun.-Nov	-1.700	0.961
Jan.-Mar	2.639	0.674	Mar.-Aug	10.035	** < 0.0001**	Jul.-Aug	2.205	0.841
Jan.-Apr	6.217	**0.017**	Mar.-Sep	-7.662	**0.003**	Jul.-Sep	0.169	1.000
Jan.-May	-6.087	**0.019**	Mar.-Oct	-9.218	**0.001**	Jul.-Oct	-1.387	0.990
Jan.-Jun	-4.783	0.087	Mar.-Nov	-9.122	**0.001**	Jul.-Nov	-1.291	0.995
Jan.-Jul	-5.192	0.054	Apr.-May	0.13	1.000	Aug.-Sep	2.374	0.780
Jan.-Aug	7.397	**0.005**	Apr.-Jun	1.434	0.988	Aug.-Oct	0.818	1.000
Jan.-Sep	-5.023	0.066	Apr.-Jul	1.025	0.999	Aug.-Nov	0.914	1.000
Jan.-Oct	-6.579	**0.011**	Apr.-Aug	-1.18	0.997	Sep.-Oct	1.556	0.978
Jan.-Nov	-6.483	**0.012**	Apr.-Sep	1.194	0.997	Sep.-Nov	1.460	0.986
Feb.-Mar	1.873	0.931	Apr.-Oct	-0.362	1.000	Oct.-Nov	-0.096	1.000

*Abbreviations*: *CI* Condition index, *T* The least mean square difference

* P* Probability at α ≤ 0.05 where the bold values indicate substantial differences

### Sexuality

*Paphia textile* lacks sexual dimorphism. Both male and female *P. textile* gonads have cream to yellow in color. The histological sections prove that it is dioecious; no hermaphroditism was observed in the examined specimens. A single gonad is located above or adjacent to the ventral muscular foot of both sexes. Gametes are discharged through an exhalant siphon, and fertilization is completed externally.

### Gonadal development stages

**Fig. 4:**
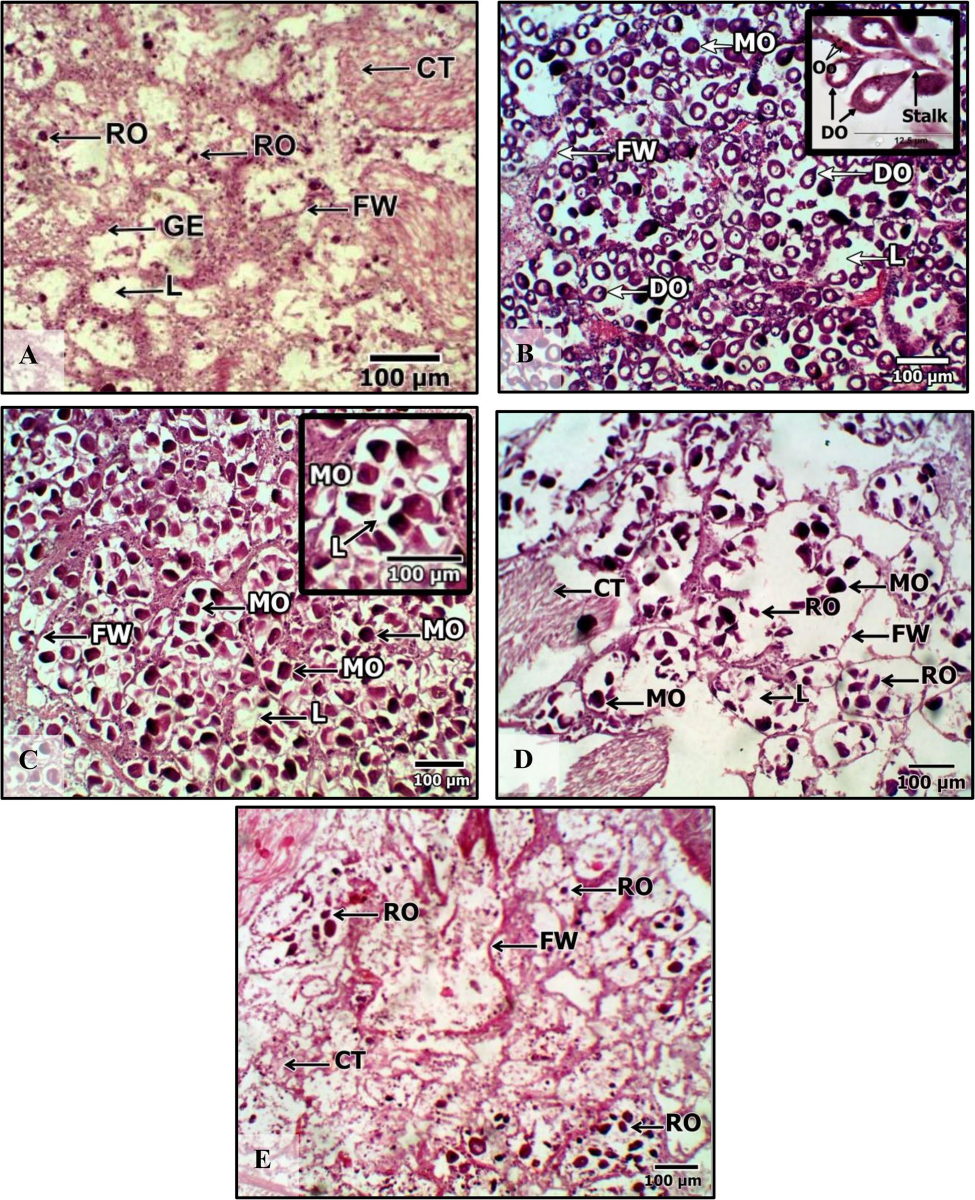
Photomicrographs of the results of female *Paphia textile's* gonadal development stages during the study period. **A** resting stage (Stage I); **B** developing stage (Stage II). **C** ripe (Stage III); **D** spawning (Stage IV). E. spent (Stage V). Abbreviations: Abbreviation: GE = Germinal epithelium; FW = follicle wall; Oo = Oogonia; DO = developing oocyte; MO = mature oocyte; RO = residual oocyte; CT = connective tissue; L = lumen

**Fig. 5:**
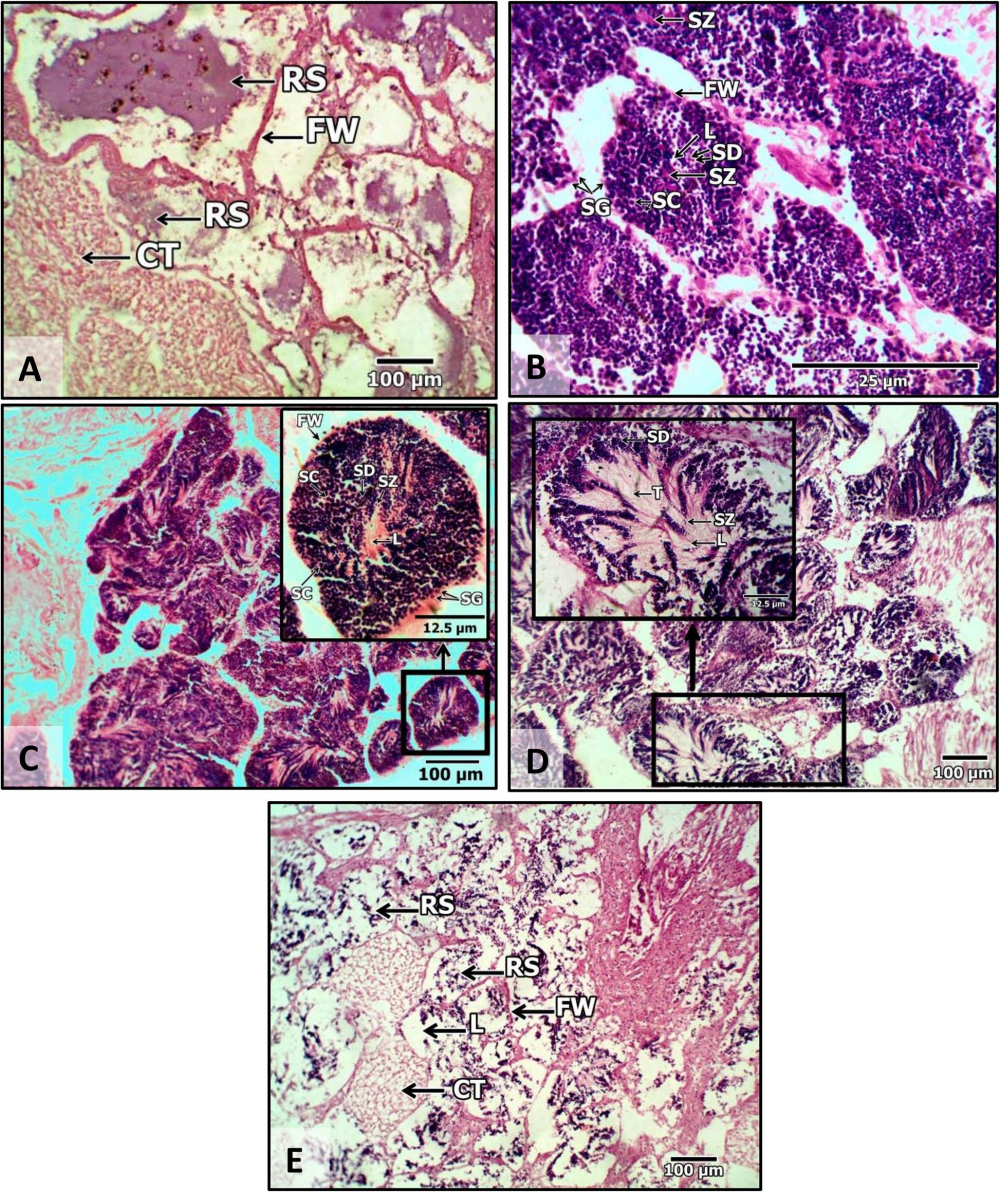
Photomicrographs of the results of male *Paphia textile's* gonadal development stages during the study period **A** resting stage (Stage I); **B** developing stage (Stage II). **C** ripe (Stage III); **D** spawning (Stage IV). **E** spent (Stage V). Abbreviation: FW = follicle wall; SG = spermatogonia, SC = spermatocyte, SD = spermatid; SZ = spermatozoa; RS = residual spermatozoa; L = lumen; CT = connective tissue; T = spermatozoa tails

The reproductive cycle of the carpet clam *P. textile* consisted of five maturity stages in both males and females. The maturity stage differentiation is based on the follicles' shape, components, internal organization, gamete type’s investigation, growth, and multiplication. A detailed differentiation description of each stage character in females and males is shown in Table 6 and figured out in Fig. 4 (females) and Fig. 5 (males). The character of the resting stage is the same in females as in males. The second and third stages have different components for both sexes. Both sexes share some common phenomena in the final two stages (IV and V). The gametes are released in stage IV, leaving the follicles relatively empty depending on the spawning power. Follicles are empty, scattered, and with broken walls during the spent stage (V). However, other components are different in both sexes.

**Table 6:** Histological characteristics of the gonadal development stages of *P. textile* collected from TL and GBL during the study period between December 2019 and November 2020

**Sexes**	**Maturation Stages**
	**Resting stage (I)**
**Both sexes**	The resting stage is also called inactive or undifferentiated. There is a trace of gonad development. There are slightly distinguishable differences in the follicles of females and males. The follicle is surrounded by germinal epithelium, and there are a variety of connective tissues between the follicles. A few residual spermatozoa and oocytes may be present (Figs. 4 and 5A)
	**Developing stage (II)**
**Female**	Oogonia are found at various developmental stages at the follicle periphery. A stalk connects the oocytes to the wall of the follicle, and they begin to fill the follicles. Some free oocytes are present in the lumen. But they constitute a smaller ratio. (Fig. 4B)
**Male**	Rounded to expanded follicles are detected. Spermatogonia proliferate and give rise to several layers of spermatocytes that are expanded toward the lumen. Few spermatids are observed, and few spermatozoa are proliferated at the lumen (Fig. 5B)
	**Ripe stage (III)**
**Female**	Follicles are filled with mature oocytes, that have fallen in the lumen. They range in shape from rounded to elongated or irregular, and their ooplasm is filled with yolk. Lumen is barely visible. Stalked oocytes at the follicle wall have nearly vanished. (Fig. 4C)
**Male**	Follicles are elongated, assuming rosette formation, and filled with spermatozoa showing their acidophilic tails as pink lines proliferate at the lumen (Fig. 5C)
	**Spawning stage (IV)** “The gametes are discharged. Depending on the degree of spawning, the follicles are relatively empty”
**Female**	Each follicle has a few free oocytes. Some follicles are empty, due to ova release. The follicle walls are disorganized. Connective tissue is present between the follicles (Fig. 4D)
**Male**	Follicles show a streaky appearance from streaming sperm. The lumina are occupied by spermatozoa, with many gaps (Fig. 5D)
	**Spent stage (V)** ''Follicles appear more disorganized with broken walls, scattered, and relatively empty''
**Female**	Connective tissue is present between the follicles. Residual oocytes are barely seen in the follicles (Fig. 4E)
**Male**	Connective tissue is present between the follicles. Some follicles were empty, but others contained residual sperm with many gaps (Fig. 5E)

*Abbreviations*: *TL* Timsah Lake and *GBL* Great Bitter Lake

### Reproductive cycle of *Paphia textile*

**Fig. 6:**
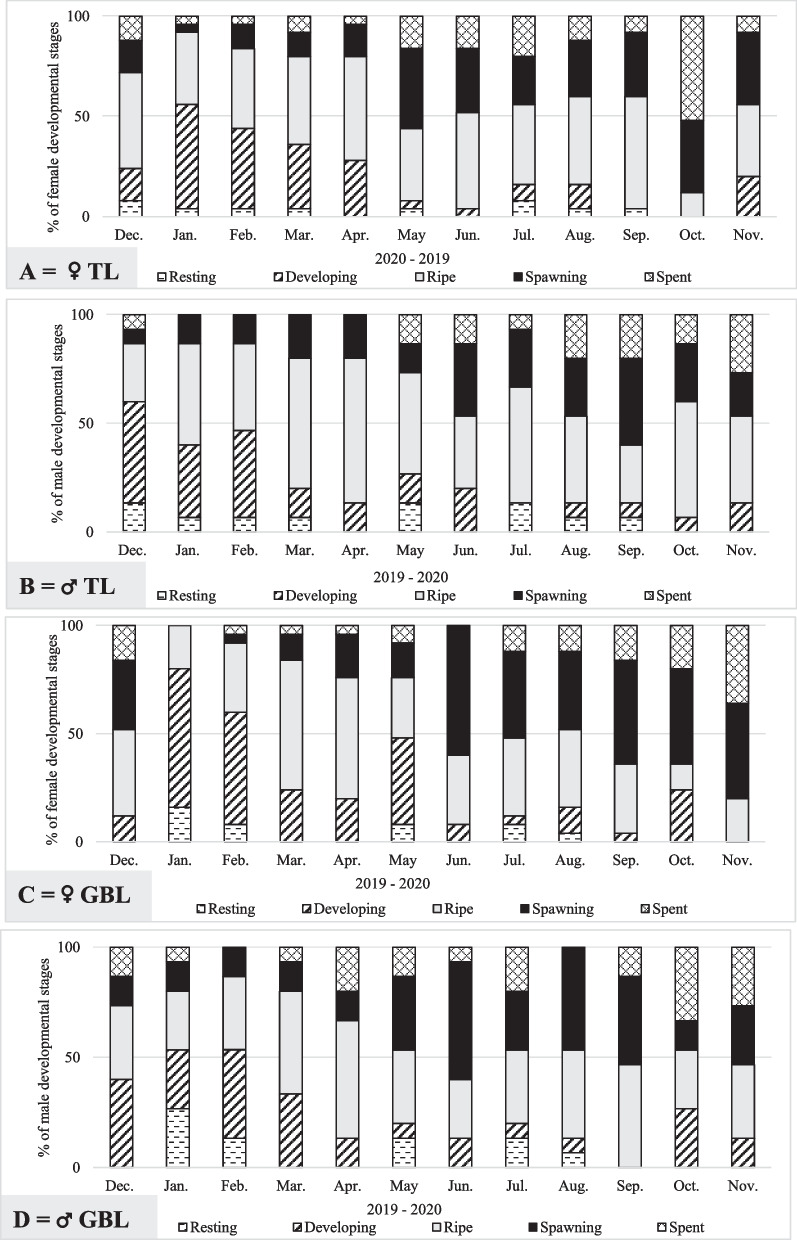
Monthly relative frequency (%) of gonadal development stages of *Paphia textile* in TL females (**A**) and males (**B**) and in GBL females (**C**) and males (**D**) from December 2019 to November 2020. Abbreviations: TL = Timsah Lake; GBL = Great Bitter Lake

The gonadal maturity stage distribution and the percentage of frequency (occurrence) revealed a wide range of monthly variation and lacked periodicity for both sexes (Fig. 6). The resting stage distributional range was narrow, and data did not locate stage I in some months (April, June, and October, Fig. 6), The highest frequency of stage I was 13.3% (TL), and 26.7% (GBL). There were only slight variations between the histograms of males and females (Fig. 6).

The developing stage (II) was highest between January and April at TL and in January and February at GBL for female clams (Fig. 6A and C). January’s highest contribution fluctuated between 52% at TL and 64% at GBL, whereas the developing stage disappeared in September and October at TL and November at GBL. The pattern of developing stages of the male clams was inconsistent throughout the study year. However, the highest contribution was detected during the early winter at TL and in winter and spring at GBL, with few exceptions (Fig. 6B and D).

The ripe females (stage III) were the most frequent in April (52%) and September (56%) at TL and during March (60%) and April (65%) at GBL (Fig. 6A and C). Ripe males were recorded year-round with a wide frequency range (Fig. 6B and D).

The ripe stage was followed by two spawning peaks (stage IV) for females at both studied sites. In TL, the first and second spawning peaks occurred in May (40%) and October and November (36%). In GBL, the two peaks were detected in June (60%) and September (48%). Male spawning stages were found throughout the year and tracked their females in both study sites (Fig. 6B and D).

The highest contribution of the completely spawned females (stage V) was observed in October (52%) and November (36%) at TL and GBL, respectively. The spent-stage males were found from May to December, with the highest contribution in November (26.7%), at TL (Fig. 6B). At GBL, the proportion of spent males (stage V) was recorded throughout the study year, except for February and August, and peaked in October (33.3%, Fig. 6D).

Gonadal development stages were found to significantly vary by month (*P* ≤ 0.001, Table 7). However, the results of the 3-way ANOVA did not reveal significant differences between the sampling areas, sexes, and the interactions among categorical factors, except for the mean data of stage III, which was significantly different between TL and GBL (*P* = 0.005, Table 7).

**Table 7:** Results of three-way ANOVA analysis based on square root transformed data of maturity stages I, II, IV, V, and raw data of stage III between the sampling locations, sexes, and within the months

Maturity stages	Source	Sum-of-Squares	df	Mean-Square	F-ratio	*P*
**Stage I**	Location	0.328	1	0.328	3.302	0.078
	Sex	0.185	1	0.185	1.868	0.181
	Month	10.994	11	0.999	10.068	** < 0.0001**
**Stage II**	Location	1.264	1	1.264	0.649	0.426
	Sex	0.001	1	0.001	0	0.986
	Month	119.277	11	10.843	5.572	** < 0.0001**
**Stage III**	Location	739.47	1	739.47	8.883	**0.005**
	Sex	103.253	1	103.253	1.24	0.273
	Month	3231.91	11	293.81	3.529	**0.002**
**Stage IV**	Location	1.043	1	1.043	1.04	0.315
	Sex	0.358	1	0.358	0.356	0.554
	Month	73.961	11	6.724	6.702	** < 0.0001**
**Stage V**	Location	0.049	1	0.049	0.03	0.864
	Sex	0.587	1	0.587	0.356	0.555
	Month	71.825	11	6.53	3.959	**0.001**

*Abbreviations*: *df* Degree of freedom, *F- ratio* F statistic

*P* Probability at α ≤ 0.05 and bold values indicate substantial differences

Results from the Tukey test (Table 8) detected that stage I data had the most significantly frequent least mean differences year-round. The distributional frequency of stage I was significantly higher in January and February than in April (T = 1.21 and 1.04, respectively Table 8). The T values were significantly higher in June, October, and November than in January and February. The data revealed significant variations in spring, April vs. May, July, and August, and May vs. June, October, and November. Further, the in-between data frequencies during the warm months were significantly different in June vs. July, August, October, and November, and in July and August vs. October and November (Table 8). The trend of monthly least mean differences in the distributional data of the other four stages was less clear. The least statistically significant differences in stage II data were observed in winter (January and February) vs. summer (July and August) and Autumn (September and November). Significant least mean differences in stage III were detected in January, March, and April vs. April, October, and October–November, respectively. The significantly different least mean stage IV data were recorded in January vs. May, summer, and autumn months, and February’s stage IV data differed considerably from June and August. The January and March least mean stage V data differed significantly from October–November and October, respectively (Table 8).

**Table 8:** Results of the posterior hoc Tukey test analysis based on transformed square root data of maturity stages of *Paphia textile* I, II, IV, V, and raw data of stage III throughout the sampling period December 2019 to November 2020 at α ≤ 0.05

Months	St. I	St. II	St. III	St. IV	St. V	Months	St. I	St. II	St. III	St. IV	St. V
	**T**	**T**	**T**	**T**	**T**		**T**	**T**	**T**	**T**	**T**
Dec.-Jan	0.62	1.39	-4.5	-1.57	-2.1	Mar.-Jul	-0.73	3.03	12.18	-1.64	-1.80
Dec.-Feb	0.451	1.388	-0.68	-0.78	-2.26	Mar.-Aug	-0.46	1.94	12.75	-2.07	-0.98
Dec.-Mar	-0.14	-0.17	15.83	-0.21	-1.47	Mar.-Sep	0.00	-3.68	-12.33	2.55	1.78
Dec.-Apr	0.59	0.9	-20.00	0.173	1.22	Mar.-Oct	-0.45	-1.74	-26.68*	1.60	3.32*
Dec.-May	0.524	-1.50	-1.00	0.963	0.11	Mar.-Nov	-0.45	-1.98	-20.50	1.82	2.85
Dec.-Jun	-0.59	-1.92	-1.85	2.65*	-0.77	Apr.-May	1.12**	-0.60	-21.00	0.79	1.32
Dec.-Jul	0.59	-3.20	3.65	1.43	0.33	Apr.-Jun	0.00	-1.02	-21.85	2.47	0.45
Dec.-Aug	-0.32	2.109	-3.08	-1.86	0.49	Apr.-Jul	1.2***	-2.30	-16.35	1.26	1.55
Dec.-Sep	-0.14	-3.85	3.5	2.34	0.31	Apr.-Aug	0.91*	-1.21	-16.93	1.69	0.73
Dec.-Oct	-0.59	-1.91	-10.9	1.39	1.85	Apr.-Sep	0.45	-2.95	-16.50	2.17	1.53
Dec.-Nov	-0.59	-2.15	-4.68	1.61	1.38	Apr.-Oct	0.00	-1.01	-30.85**	1.21	3.07
Jan.-Feb	0.17	0.00	-3.83	-0.79	0.16	Apr.-Nov	0.00	-1.25	-24.68*	1.44	2.60
Jan.-Mar	-0.76	-1.56	20.33	1.36	0.63	May-Jun	1.12**	0.43	0.85	-1.68	0.87
Jan.-Apr	1.21***	2.29	-24.50*	-1.74	-0.88	May-Jul	-0.07	1.71	-4.65	-0.47	-0.23
Jan.-May	-0.10	-2.88	3.50	2.53*	2.20	May-Aug	0.21	0.61	-4.08	-0.90	0.60
Jan.-Jun	-1.21***	-3.31	2.65	4.22***	1.33	May-Sep	-0.66	-0.65	4.50	1.38	0.21
Jan.-Jul	-0.03	-4.59**	8.15	3.00*	2.43	May-Oct	-1.12**	-0.41	-9.85	0.42	1.74
Jan.-Aug	0.30	3.50*	-7.58	-3.43**	-1.61	May-Nov	-1.12**	-0.65	-3.68	0.65	1.28
Jan.-Sep	-0.76	-5.24***	8.00	3.91***	2.41	Jun.-Jul	-1.18***	1.28	-5.50	1.22	-1.10
Jan.-Oct	-1.21***	-3.29	-6.35	2.96*	3.95*	Jun.-Aug	-0.91*	0.19	-4.93	0.78	-0.28
Jan.-Nov	-1.21***	-3.53*	-0.18	3.18**	3.48*	Jun.-Sep	0.45	-1.93	5.35	-0.31	1.08
Feb.-Mar	-0.59	-1.56	16.50	0.57	0.79	Jun.-Oct	0.00***	0.02	-9.00	-1.26	2.62
Feb.-Apr	1.04**	2.29	-20.68	-0.95	-1.04	Jun.-Nov	0.00***	-0.22	-2.83	-1.04	2.15
Feb.-May	0.07	-2.88	-0.33	1.74	2.36	Jul.-Aug	0.27	-1.09	0.58	-0.43	0.82
Feb.-Jun	-1.04**	-3.31	-1.18	3.43**	1.49	Jul.-Sep	-0.73	-0.65	-0.15	0.91	-0.02
Feb.-Jul	0.14	-4.59**	4.33	2.21	2.59	Jul.-Oct	-1.18***	1.30	-14.50	-0.05	1.52
Feb.-Aug	0.13	3.50*	-3.75	-2.64*	-1.77	Jul.-Nov	-1.18***	1.06	-8.33	0.18	1.05
Feb.-Sep	-0.59	-5.24***	4.18	3.12	2.57	Aug.-Sep	-0.46	-1.74	0.43	0.48	0.80
Feb.-Oct	-1.04**	-3.29	-10.18	2.17	4.11**	Aug.-Oct	-0.91*	0.20	-13.93	-0.48	2.34
Feb.-Nov	-1.04**	-3.53*	-4.00	2.39	3.64*	Aug.-Nov	-0.91*	-0.04	-7.75	-0.25	1.87
Mar.-Apr	0.45	0.73	-4.18	-0.39	-0.25	Sep.-Oct	0.45	-1.94	14.35	0.96	-1.54
Mar.-May	0.66	-1.33	-16.83	1.18	1.57	Sep.-Nov	0.45	-1.70	8.18	0.73	-1.07
Mar.-Jun	0.45	1.75	17.68	-2.86*	-0.70	Oct.-Nov	0.00	0.20	-6.18	0.73	0.47

*Abbreviations*: *St.* Maturity stage, *T* The least mean square difference, the bold values indicate substantial differences

* = *P* < 0.05, ** = *P* < 0.005, *** = *P* < 0.0005

The relationships between the data of maturity stages and the measured environmental factors differed in sign and magnitude, as indicated by the Pearson correlation analysis (Table 9). The temperature of the water affected the distribution of stages I and II negatively (*r* = -0.69 and -0.72 at *P* = 0.003 and 0.002, respectively), and had a mildly significant positive effect on stages IV and V (*r* = 0.58 and 0.57, *P* = 0.018 and 0.019, respectively). In contrast to the temperature effect, salinity correlated significantly positively with data from the first two stages, since their r values were 0.572 (*P* = 0.02) and 0.866 (*P* < 0.0001), respectively. Relatively high salinity negatively affected the last two stages (*r* = -0.743 and -0. 551, at *P* = 0.001 and 0.027, respectively). The effect of the dissolved oxygen was confined to stage I with relatively strong positive significant relationship (*r* = 0.68 at *P* = 0.004). Surprisingly, chlorophyll *a* data revealed strong negative (*r* = -0.799 at *P* < 0.0001) and positive (*r* = 0.815 and *P* < 0.0001) correlations with the first and last stages, respectively. However, the pH data did not reveal significant correlations with different maturity stages.

**Table 9:** Results of Pearson correlation analysis between squared root transformed data of the *Paphia textile* maturity stages I, II, IV, and V, and raw data from Stage III and the measured environmental parameters in the studied locations and throughout the sampling period

Environmental Parameter	Stage I		Stage II		Stage III		Stage IV		Stage V	
	**r**	***P***	**r**	***P***	**r**	***P***	**r**	***P***	**r**	***P***
**Temperature**	-0.690	**0.003**	-0.721	**0.002**	0.276	0.300	0.580	0.**018**	0.578	**0.019**
**pH**	-0.385	0.141	0.290	0.276	0.098	0.719	-0.351	0.183	0.137	0.613
**Salinity**	0.572	**0.021**	0.866	** < 0.0001**	-0.251	0.348	-0.743	**0.001**	-0.551	**0.027**
**DO**	0.680	**0.004**	0.316	0.233	-0.240	0.370	-0.187	0.488	-0.464	0.070
**Chlorophyll *a***	-0.799	** < 0.0001**	-0.457	0.075	-0.395	0.130	0.447	0.083	0.815	** < 0.0001**

*Abbreviation*: *DO* Dissolved oxygen, *r* Correlation coefficient

*P* Probability at α ≤ 0.05. The bold values indicate significant correlation

### Clam size at first sexual maturity

**Fig. 7:**
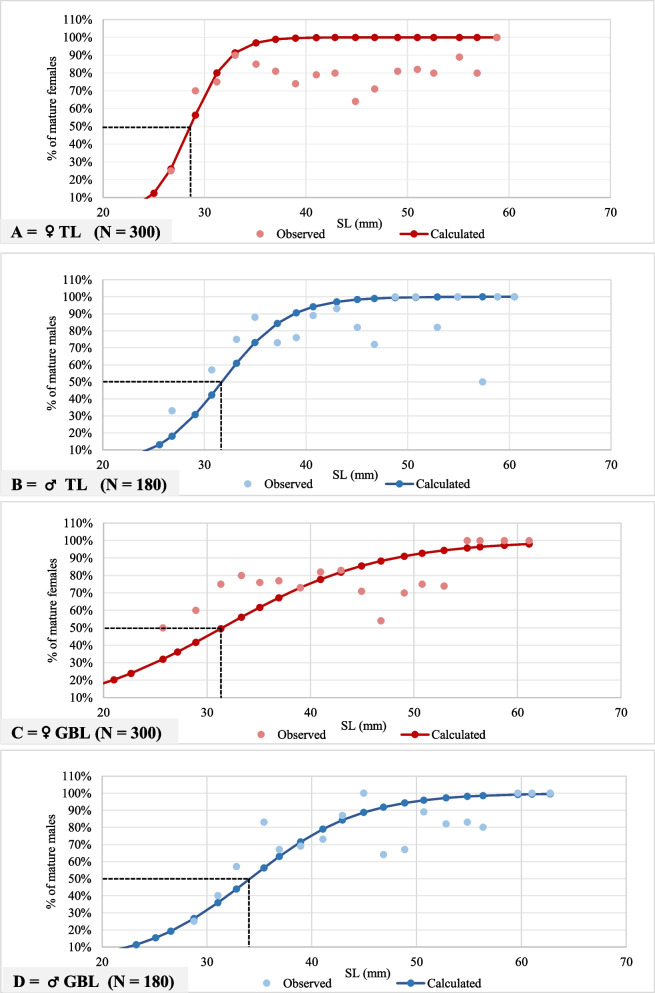
Relationship between shell length (mm) and % of mature female and male *Paphia textile* collected from TL (**A**: females, **B**: males) and GBL (**C**: females, **D**: males) during the study period. The size of 50% maturity is demonstrated (N = number of clams investigated). Abbreviations: SL = shell length; TL = Timsah Lake; GBL = Great Bitter Lake

The calculated mature clam shell length sizes (mm) at SM_50_ were 28.60- and 31.70-mm SL for females and males in TL, respectively. At GBL, SM_50_ was 31.50 mm for females, and 34.10 for males (Fig. 7), indicating the size of mature individuals was smaller at TL than at GBL. The observed results of the microscopic examination revealed that the length of the shell of mature clams was between 27.55 mm for females and 25.60 mm for males in TL. At GBL, the SL values at the maturity were 25.55 mm and 27.70 mm for females and males, respectively.

### Gonad index

**Fig. 8:**
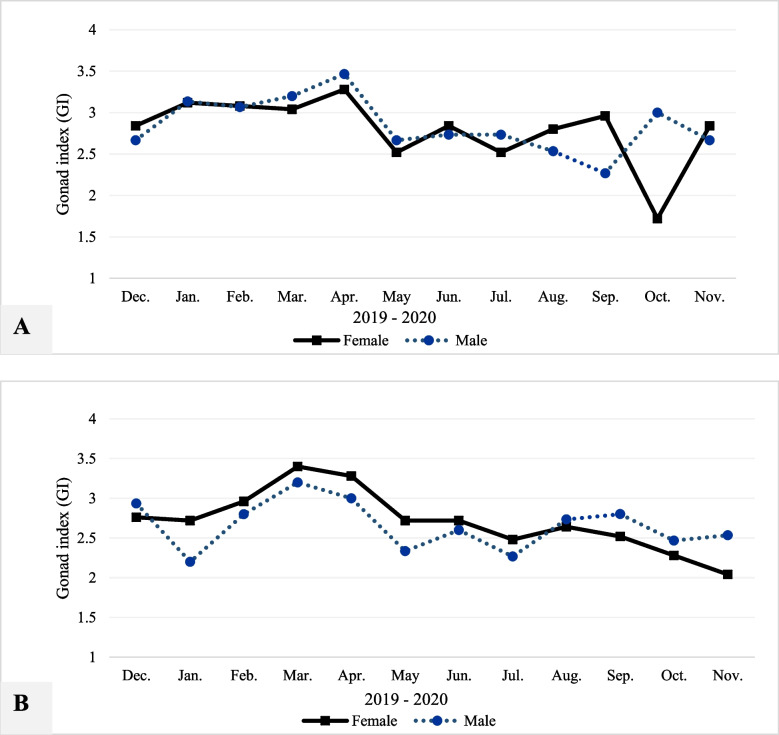
Gonad index results of *Paphia textile* in TL (**A**) and GBL (**B**) during the study period between December 2019 and November 2020. Abbreviations: TL = Timsah Lake; GBL = Great Bitter Lake

Gonad index results revealed fluctuation during the sampling period between December 2019 and November 2020 at TL (Fig. 8A) and GBL (Fig. 8B). The highest TL scores were detected in April (3.47 for males and 3.28 for females), and the lowest values were recorded in September (2.27 for males) and in October (1.72 for females). Furthermore, female values were slightly higher in GBL than at TL and vice versa for the male data. However, mean GI data at TL was not significantly different from the data at GBL (Table 10). The scoring of the GI data varied markedly among months (F-ratio = 4.423 at *P* < 0.0001; Table 10). The Tukey analysis indicated that GI data were significantly different in March vs. July, October, and November (*P* = 0.023, 0.003, 0.030, and 0.027, respectively, Table 11). Mean GI data in April was significantly different from July and October (*P* = 0.012 and 0.002, respectively Table 11).

**Table 10:** Results of three-way ANOVA analysis of gonad index values of *Paphia textile* among different study locations throughout the study months between December, 2019 and November, 2020

Source	SS	df	MS	F-ratio	*P*
**Sex**	0.000	1	0.000	0.002	0.965
**Location**	0.230	1	0.230	3.317	0.077
**Months**	3.368	11	0.306	4.423	** < 0.0001**

*Abbreviations*: *SS* Sum squares, *df* Degree of freedom, *MS* Mean square, *F-ratio* F statistic

* P* Probability at α ≤ 0.05 and bold values indicate substantial differences

**Table 11:** Results of Tukey test of gonad index values of *Paphia textile* throughout the study months between December 2019 and November 2020

Months	GI		Months	GI		Months	GI	
	**T**	***P***		**T**	***P***		**T**	***P***
Dec.-Jan	-0.007	1.000	Feb.-Apr	-0.280	0.929	Apr.-Nov	-0.737	**0.016**
Dec.-Feb	0.177	0.998	Feb.-May	-0.417	0.532	May-Jun	-0.163	0.999
Dec.-Mar	0.410	0.558	Feb.-Jun	-0.255	0.962	May-Jul	0.060	1.000
Dec.-Apr	-0.458	0.397	Feb.-Jul	-0.477	0.336	May-Aug	-0.115	1.000
Dec.-May	-0.240	0.975	Feb.-Aug	0.302	0.887	May-Sep	0.078	1.000
Dec.-Jun	0.078	1.000	Feb.-Sep	-0.340	0.792	May-Oct	-0.192	0.996
Dec.-Jul	-0.300	0.893	Feb.-Oct	-0.610	0.084	May-Nov	-0.040	1.000
Dec.-Aug	0.125	1.000	Feb.-Nov	-0.457	0.397	Jun.-Jul	0.222	0.986
Dec.-Sep	-0.163	0.999	Mar.-Apr	-0.047	1.000	Jun.-Aug	0.047	1.000
Dec.-Oct	0.433	0.480	Mar.-May	-0.650	0.051	Jun.-Sep	-0.085	1.000
Dec.-Nov	-0.280	0.929	Mar.-Jun	0.488	0.308	Jun.-Oct	-0.355	0.746
Jan.-Feb	-0.185	0.997	Mar.-Jul	0.710	**0.023**	Jun.-Nov	-0.202	0.993
Jan.-Mar	0.417	0.532	Mar.-Aug	0.535	0.195	Jul.-Aug	-0.175	0.998
Jan.-Apr	-0.465	0.374	Mar.-Sep	-0.573	0.130	Jul.-Sep	0.137	1.000
Jan.-May	-0.233	0.980	Mar.-Oct	-0.843	**0.003**	Jul.-Oct	-0.133	1.000
Jan.-Jun	-0.070	1.000	Mar.-Nov	-0.690	**0.030**	Jul.-Nov	0.020	1.000
Jan.-Jul	-0.292	0.907	Apr.-May	-0.698	**0.027**	Aug.-Sep	-0.038	1.000
Jan.-Aug	0.118	1.000	Apr.-Jun	-0.535	0.195	Aug.-Oct	-0.308	0.877
Jan.-Sep	-0.155	0.999	Apr.-Jul	-0.757	**0.012**	Aug.-Nov	-0.155	0.999
Jan.-Oct	0.425	0.506	Apr.-Aug	-0.583	0.116	Sep.-Oct	0.270	0.994
Jan.-Nov	-0.272	0.940	Apr.-Sep	-0.620	0.075	Sep.-Nov	0.117	1.000
Feb.-Mar	0.233	0.980	Apr.-Oct	-0.890	**0.002**	Oct.-Nov	-0.153	0.999

*Abbreviations*: *GI* Gonad index, *T* The least mean square difference

* P* Probability at α ≤ 0.05 and bold values indicate substantial differences

## Discussion

This work is complementary to a previous study [1]. Both aim to give a detailed investigation of the reproductive cycle of *P. textile.* It is one of the most commercial, edible, and highly productive clams along the Suez Canal [5]. The *P. textile* average shell length ranged from 41.60 ± 7.52 mm (TL) to 43.69 ± 7.62 mm (GBL), while the average total wet weight accounted for 7.92 ± 4.86 g and 9.32 ± 5.15 g at TL and GBL, respectively [1]. Reproductive studies are essential to monitor future hatchery development management [39]. Our discussion will focus on the most important results that achieve the target of this study. However, the significant differences in some measured environmental factors herein would interpret the results.

The seasonal variation of seawater temperatures across the Suez Gulf and its cities was followed by a corresponding seasonal distribution of air temperatures [40]. The monthly, seasonally, and yearly variations in air and sea temperature are the result of climate changes, latitude, longitude, wind, waves, and the ecosystem depth [41]. The significantly higher salinity values found at GBL than at TL are due to the historical origin of GBL, as GBL was a massive salt-flat lake before the construction of the Suez Canal in 1869 [42, 43]. The lower the temperature, the higher the solubility of the dissolved gases [41]. A significantly higher concentration of the dissolved oxygen was detected in the winter of 2020 than in the spring and autumn (Table 1, Fig. 2D), but surprisingly, summer had the highest significant values, which disagrees with Khedr ‘s study [41]. Several factors control the surface sea oxygen solubility; atmospheric oxygen level, organic productivity, and ocean current [44]. Letshele et al. [45] suggested the role of a high evaporation rate in increasing oxygen solubility. Lake Timsah is well known for its eutrophic characteristics [40]. Some studies documented the inverse relationships between oxygen concentrations and eutrophication [46]. The pH values herein were within the recommended range for fish and the maximum marine life productivity, ranging between 6.5 and 8.5 [47]. The significantly higher chlorophyll *a* concentration at TL than at GBL is probably due to the hypersaline oligotrophic conditions at the latter [48].

The bias in sex ratios in most living organisms is associated with the dominance of unfavorable environmental conditions, climate change, and temperature rise [49–53]. Our study detected significant male-biased ratios (Table 2) compared to female-biased ratios of *P. textile* from the Philippines waters in research conducted more than 13 years ago [26]. Male skewed ratios in Egyptian Suez Canal coasts may indicate female sensitivity to some ecological issues. Some studies have documented that TL has suffered from eutrophication [28]. Human activities have put the Suez Gulf at risk, and it is nutrient-limited [41]. Further, fluoride content in soft and shell tissues of *P. textile* from one region of the Canal cities, Ismailia, was the highest compared to other Egyptian Mediterranean coasts and ranged between 0.7 and 0.8 mg/g [8]. The fluoride concentration in El- Said's study was found to affect both genders, but it may have an indirect effect on sex ratios. A microscopic examination revealed that mature clams vary between 27.55 mm (female) and 25.60 mm (male) at TL, and between 25.55 mm and 27.70 mm at GBL (Fig. 7). In this work and our previous study [1], male shell sizes were approximately 3.5 mm larger than female shell sizes. In addition, we found that most large clams were males. The cause behind the male-biased ratio could be the high female mortality after spawning, as was reported for *Chlamys islandica* and *Mytilus trossulus* [54, 55]. The samples of *P. textile* specimens before sorting contain some dead individuals, which were excluded. In females, gamete formation requires significantly more energy than in males, because females consume energy first for growth, then for gamete development [56]. The higher the water temperature, the more male-biased the sex ratio is [53]. The food available to most Bivalvia determines its sex ratio [56]. This bias could be caused by predation on *Paphia textile* [57], since some predators may prefer to open smaller clams, or the female shell might be easier to crack than the male shell, a factor that needs further investigation. Despite numerous explanations for male-biased sex ratios, our study concluded that no single factor is sufficient. In some ways, anthropogenic stressors may be responsible for the disparity in the sex ratio.

The CI indicated the flesh and shell weight changes over the study period [58]. The CI attained its highest significant value during late winter (January–February) and early spring (March 2020, Fig. 3, Table 5), indicating that the carpet clam was in its pre-spawning and ripe stages because a high portion of specimen mass was gonadal tissue, a finding that agreed with Nottingham and White [59]. A study by Singh [60] correlated positively between the high CI and the pre-spawning peak of the gonad index. The predominance of the resting developmental stage at TL in October could be the reason behind the lowest CI in this month (Fig. 6), as a decrease in CI would capture an increase in immature individuals. Bernardes [61] documented a direct relationship between CI and mature animals.

Marine mollusks have dioecious functionality [62], and most members of Bivalvia: Veneridae lack hermaphroditic characteristics [63–65]. Recently, hermaphroditism was detected in some species of the family Veneridae in tropical waters [66, 67], while other species revealed sequential hermaphroditism [68]. Hermaphroditism, or sex reversal, is an adaptation mechanism to overcome stressful conditions [56]. However, none of the investigated specimens revealed hermaphrodism in the current study, which agrees with earlier studies [6, 26, 69]. Our findings indicate that the gene expression in *P. textile* could be strong enough to overcome unfavorable conditions or that the anthropogenic stressors in our studied locations are not severe enough to force *P. textile* to attain hermaphroditism. The male and female colors of the gonads are the same, creamy to yellow, which hinders their identification from a morphological point of view. Therefore, the smear observations and histological studies were mandatory to differentiate between sexes and identify the maturity stages.

The reproductive cycle was classified into five maturity stages based on the histological investigation, follicle appearance, and the developmental components of each maturity stage (Table 6). Our findings agreed with many studies [12, 70]. However, the maturity stages were not sequential or consecutive, as several developmental stages occurred simultaneously on a monthly basis throughout the sampling year. Lacking periodicity and the year-round *P. textile* reproduction is a common phenomenon in tropics and subtropics regions [17, 71] and is probably attributed to the dominance of warm water most of the year. However, the highest frequencies of maturity stages I, II, III, and V peaked during winter, spring, and autumn (Fig. 6), a finding which could indicate that the most favorable temperature for *P. textile* to spawn is within 20 ^◦^C and 30 ^◦^C, and temperatures higher than 30 ^◦^C could impact the rate of gonad development [10]. The highest significantly different autumn and spring temperatures exceeded 35 ^◦^C and could inhibit spawning in subsequent summer months (Fig. 2A, Table 1). The da Costa et al.’s study [10] concluded that the ideal temperature for gonad development in European waters ranged between 18 to 20 ^◦^C and exceeded twenty-two ^◦^C in some regions. As a result of significant differences in least-square means between January and February vs. the rest of the year, as well as spring and, to some extent, autumn vs. summer (Table 8), maturity stages’ frequency tended to be higher during winter, spring, and autumn. For future mariculture of *P. textile,* winter and spring are the best hatchery seasons, as the current study revealed, and caution must be considered to keep temperatures within the above range. Some studies in tropical waters indicate that the studied Bivalvia species attained the highest gamete development during winter and spring, whereas it was at a minimum in summer and autumn [72]. The stage V (spawning stage) peaks synchronized the timing of other stages except for the June peak at GBL, which could be due to the high female percentage during this month (the sex ratio was female-biased). Lacking male–female synchronization (Fig. 6) could be due to the high cost of female gametocyte development and the male-biased sex ratio. However, data is not significantly different between both sexes (Table 7).

The significant response of clam maturity stages to the surrounding environmental conditions varied in sign and magnitude (Table 9) may indicate that changes in gonadal development stages are due to the fluctuation of temperature, chlorophyll *a*, salinity, and, to some extent, dissolved oxygen throughout the year. Further, it could indicate the resilience of *P. textile* to significant differences in salinity and chlorophyll *a* values between the TL and GBL (Table 1, Fig. 2). Our results disagreed with Ilano et al. [26] conclusion for the same species in the Philippines’ waters which found no significant correlations between the gonad cycle vs. temperature and salinity due to the lacking of fluctuations of these factors between dry and rainy seasons. Vázquez [73] concluded that the response of gametogenic stages to extreme changes in salinity and temperature varied with the time of year, and temperatures greater than 32 ^◦^C could provoke changes in the gonadal cycle. Others stated that clams held at low temperatures displayed an advanced reproductive cycle compared to those kept at high temperatures [74].

The *P. textile* shell size at SM_50_ (Fig. 7) in the Egyptian waters is smaller than the sizes of three Veneridae species, *Paphia textilis, Paphia undulata,* and *Meretrix meretrix*, that inhabited the Philippines and Vitnamean coasts. However, all attained maturity at shell length ~ 40 mm or slightly longer [26, 27, 75]. The current Egyptian *Paphia* species, herein, is larger than *Venus verrucosa* (25.8 mm in the Adriatic Sea) and *Katelysia* spp (23.2 mm in South Australian waters), both are members of the family Veneridae [24, 76]. Family Veneridae displayed a wide range of shell lengths at the first maturity ranging from 10 mm to ≥ 50 mm [77]. Factors that cause the temporal and spatial variations in the maturity size (SM_50_) are numerous; they could be due to the prevailing environmental conditions, maternal care of offspring [78], or due to natural habitat variability of the species [79]. The estimation of SM_50_ aimed to detect which clam size should be prohibited from fishing [80]. Our study recommends *P. textile* fishing must be prohibited at dimensions less than 28.60 mm (♀) and 31.70 mm (♂) at TL and 31.50 mm (♀) and 34.10 mm (♂) at GBL for better natural resources management.

The gonad index's significant monthly variations (Table 10) provide a tool to monitor the reproductive cycle development of *P. textile* in the Egyptian waters of the Suez Canal. The highest GI significant values in April and March for both sexes at TL and GBL, respectively, matched stage III, where more than 45% of the population at both lakes accounted for ripe individuals (Fig. 6). However, the lowest GI values were frequently repeated in several months (Fig. 8) attained its minimum in January, May, July, and October. The timing of the low GI scoring is met with the high frequencies of resting, spawning, and spent stages, where the gonads are either undifferentiated or spawned (Fig. 6). Lagade [81] found that the GI increased as the stage of gonad maturity increased, and the scores decreased during the spawning and resting stages [77].

The current study revealed some evidence of spatial variability between the studied Egyptian lakes of the Suez Canal. The CI values were not synchronized between TL and GBL in some months; most of the maturity stages showed a one to two months delay in stages II, III, IV, and V at TL than at GBL. The sizes at maturity were relatively longer at GBL than at TL, female GI has slightly higher values in some months at GBL than at TL, and vice versa for the male GI values (Figs. 7 and 8). However, none of this evidence differentiates significantly between the two sites except for the ripe stage (III), indicating that the reproductive cycle does not support the study’s secondary aim of being a tool for spatial variation. Many factors could illustrate our findings, such as the nonsignificant variation in temperature between sites, and lacking freshwater sources. Further, the two sites are located within the same latitude (Fig. 1) with the same day length, and the sampling scale between the sites is relatively larger than 10 km. Gadomski and Lamare [82] concluded that the spatial variability in *Paphies ventricosa* in New Zealand waters was related to the cold vs. warm waters, saline vs. fresh waters, and latitudinal differences between their sampling sites. However, a recent study discussed causes of the similar reproductive cycle pattern of *Ensis magnus* in Northwest Spain with a one-month delay in the advanced maturity stages among sites, which could be due to the prolonged cold water temperatures with high salinity fluctuations [83].

## Conclusion

*Paphia textile* (Gmelin 1791), or its recent name *Paratapes textilis* (Gmelin 1791), inhabits the Egyptian waters of the Red Sea. The male-biased ratio throughout the sampling year 2019–2020 resulted from many factors. Most importantly, the unsuitable environmental condition appears to be caused by anthropogenic stressors, which are likely to be the source of this bias. The species had the highest CI during the winter and spring, indicating pre-spawning and ripened stages. The reproductive cycle consisted of five non-sequential or consecutive maturity stages and lacked periodicity, which is probably a due to the prevalence of warm water that ranged from 20 ^◦^C to more than 35 ^◦^C in the study area. The reproduction cycle continues throughout the year. The study recommended that the best temperature for clam aquaculture is within the range of 20 ^◦^C to relatively less than or equal to 30 °C to avoid temperature inhibition of the gametes' activities. Temperature, salinity, and chlorophyll *a* drove the development of maturity stages, as illustrated by the development response differing significantly in sign and magnitude. The female SM_50_ ranged between 28.60 mm and 31.50 mm, whereas male SM_50_ varied from 31.70 mm and 34.10 mm in Egyptian waters. Fishing prohibition for *Paphia* species is recommended at sizes less than 32 mm at TL and 35 mm at GBL for better natural resources management. The GI was a mirror for the reproductive cycle development because its highest and lowest values indicate the timing of the ripe stage versus the resting, spawning, and spent stages. The similarity in *P. textile* distribution between the TL and GBL along the Suez Canal, despite their significant variations in salinity and chlorophyll* a*, could be due to the prevailing warm water and saline water dominance.

### Supplementary Information

**Additional file 1: Appendix 1.** Monthly length-frequency distribution of *P. textile* males and females in TL and GBL from December, 2019 to November, 2020. Abbreviations: TL = Timsah Lake; GBL = Great Bitter Lake.

**Additional file 2: Appendix 2.** Schematic representation of the carpet clam *P. textile* shell measurements: SL: Shell length; SH: Shell height; SI: Shell inflation. (SL= 45 mm), published in Farghaley et al., 2022.

## Data Availability

The data that support the findings of this study are available from the first author, upon reasonable request.
