# Supplementary material for: Reproductive studies on the carpet clam Paphia textile (Paratapes textilis) (Gmelin 1791) (Family: Veneridae): a guide of aquaculture management along the Egyptian coasts of the Red Sea and Suez Canal
Source: BMC Zool. 2023 Sep 7;8:18. doi: 10.1186/s40850-023-00179-4 (PMC10485947; doi:10.1186/s40850-023-00179-4)
Supplement: Supplementary file 1 — Additional file 1: Appendix 1. Monthly length-frequency distribution of P. textile males and females in TL and GBL from December, 2019 to November, 2020. Abbreviations: TL = Timsah Lake; GBL = Great Bitter Lake. [file 40850_2023_179_MOESM1_ESM.pdf]

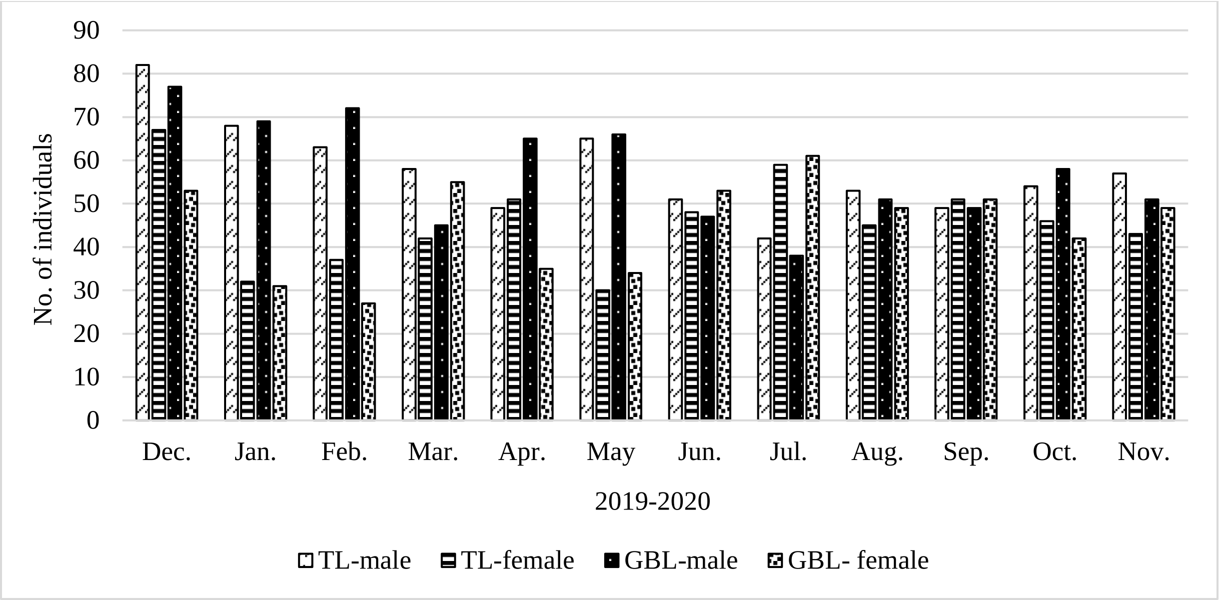

Appendix 1. Monthly length-frequency distribution of *P. textile* males and females in TL and GBL from December, 2019 to November, 2020. Abbreviations: TL = Timsah Lake; GBL = Great Bitter Lake.
