# Supplementary material for: Reproductive studies on the carpet clam Paphia textile (Paratapes textilis) (Gmelin 1791) (Family: Veneridae): a guide of aquaculture management along the Egyptian coasts of the Red Sea and Suez Canal
Source: BMC Zool. 2023 Sep 7;8:18. doi: 10.1186/s40850-023-00179-4 (PMC10485947; doi:10.1186/s40850-023-00179-4)
Supplement: Supplementary file 2 — Additional file 2: Appendix 2. Schematic representation of the carpet clam P. textile shell measurements: SL: Shell length; SH: Shell height; SI: Shell inflation. (SL= 45 mm), published in Farghaley et al., 2022. [file 40850_2023_179_MOESM2_ESM.pdf]

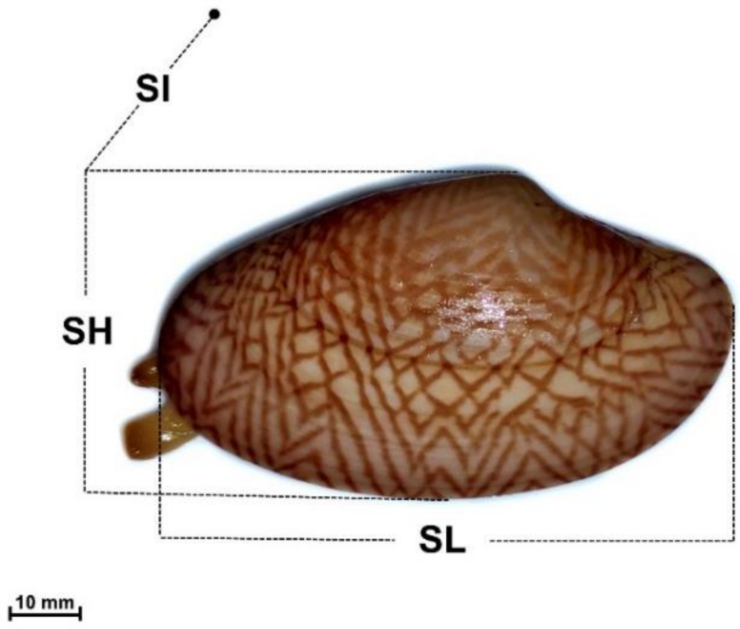

Appendix 2. Schematic representation of the carpet clam *P. textile* shell measurements: SL: Shell length; SH: Shell height; SI: Shell inflation. (SL= 45 mm), published in Farghaley et al., 2022
